# Targeting Signaling Excitability in Cervical and Pancreatic Cancer Cells Through Combined Inhibition of FAK and PI3K

**DOI:** 10.3390/ijms26073040

**Published:** 2025-03-26

**Authors:** Chao-Cheng Chen, Suyang Wang, Jr-Ming Yang, Chuan-Hsiang Huang

**Affiliations:** 1 Department of Pathology, Johns Hopkins Medical Institutions, Baltimore, MD 21231, USA; jimmy10421@gmail.com (C.-C.C.);; 2 Department of Cell Biology, School of Medicine, Johns Hopkins University, Baltimore, MD 21205, USA; 3 Center for Cell Dynamics, School of Medicine, Johns Hopkins University, Baltimore, MD 21205, USA

**Keywords:** RTK/Ras/PI3K/ERK signaling network, excitability, drug combination, targeted therapy, synergy

## Abstract

The Ras/PI3K/ERK signaling network is frequently mutated and overactivated in various human cancers. Focal adhesion kinase (FAK) is commonly overexpressed in several cancer types and has been implicated in treatment resistance mechanisms. A positive feedback loop between Ras, PI3K, the cytoskeleton, and FAK was previously shown to drive Ras signaling excitability. In this study, we investigated the effectiveness of targeting Ras signaling excitability by concurrently inhibiting FAK and PI3K in cervical and pancreatic cancer cells, which depend on activation Ras/PI3K signaling. We found that the combination of FAK and PI3K inhibitors synergistically suppressed the growth of cervical and pancreatic cancer cell lines through increased apoptosis and decreased mitosis. PI3K inhibitors alone caused only a transient suppression of downstream AKT activity and paradoxically increased FAK signaling in cancer cells. The addition of an FAK inhibitor effectively counteracted this PI3K-inhibitor-induced FAK activation. Furthermore, PI3K inhibitors were found to activate multiple receptor tyrosine kinases (RTKs), including insulin receptor, IGF-1R, EGFR, HER2, HER3, AXL, and EphA2. Taken together, our results suggest that FAK inhibition is necessary to counteract the compensatory RTK activation induced by PI3K inhibitors, thereby achieving more effective suppression of cancer cell growth. These findings highlight the therapeutic potential of combined FAK and PI3K inhibition in cancer treatment.

## 1. Introduction

The signaling network involving Ras GTPases and their downstream effectors, including the phosphoinositide 3-kinase (PI3K) and mitogen-activated protein kinase (MAPK)/extracellular signal-regulated kinase (ERK) pathways, regulates diverse cellular processes such as proliferation, differentiation, apoptosis, metabolism, protein synthesis, and motility [1]. Aberrant activation of this network, through mutations or overexpression, is frequently observed in human cancers [2]. For instance, 10–36% of cervical cancers harbor activating mutations in *PIK3CA*, which encodes the catalytic subunit of PI3Kα [3,4,5,6], while KRAS mutations are present in approximately 8% of non-squamous cell carcinomas of the cervix [7]. The MAPK/ERK pathway has also been implicated in cervical cancer pathogenesis [8,9]. Although clinical trials of PI3K pathway inhibitors have shown promise in stabilizing metastatic cervical cancer, responses have been variable [10,11]. In pancreatic ductal adenocarcinoma (PDAC), KRAS mutations are present in over 90% of tumors [12,13]. Additionally, some hereditary forms of pancreatic cancer are linked to mutations affecting the post-translational modification of Ras GTPases [14]. In KRAS wild-type PDAC, activation of this signaling network can occur through alternative mechanisms, such as BRAF mutations or RTK fusions [15].

Recent studies on the spatiotemporal dynamics of Ras/PI3K/ERK signaling at the cellular level have revealed features characteristic of excitable systems. For instance, Ras and PI3K activities propagate as self-organized waves in various cell types, including *Dictyostelium*, neutrophils, fibroblasts, and epithelial cells [16,17,18,19,20,21,22,23,24], whereas ERK exhibits pulsatile activation in individual cells and can propagate as waves across cell populations [25,26,27,28,29,30]. Furthermore, when stimulated by growth factors or chemoattractants, the activation of this signaling network displays all-or-none characteristics and refractoriness, hallmarks of excitable behavior [21,22,23,31,32,33]. Our previous study demonstrated that pulsatile ERK activity is driven by localized Ras activation at protrusions, which are stochastically generated by a positive feedback loop involving Ras, PI3K, actin, and focal adhesion kinase (FAK) [23]. Chemical and mechanical stimuli can modulate the frequency of protrusions and ERK pulses by entering the feedback loop at different points. This excitability property enables the Ras signaling network to integrate diverse stimuli in the regulation of cell proliferation. Additionally, we found that oncogenic mutations enhance the excitability of the Ras/PI3K/ERK signaling network [22,23].

In this study, we tested the hypothesis that inhibiting the molecular mechanism underlying Ras signaling excitability can effectively suppress cancer cell growth, focusing on the positive feedback loop involving Ras, PI3K, actin, and FAK [23]. Given the potential for redundancy in signaling pathways, we reasoned that effective inhibition of excitability would require simultaneous targeting of multiple proteins. Supporting this concept, multipoint inhibition within the same signaling pathway has been shown to be more effective than targeting a single node [34]. To evaluate this, we investigated whether the combined inhibition of PI3K and FAK synergistically reduces cancer cell viability. This approach was chosen because: (1) small-molecule inhibitors for these kinases are readily available; (2) FAK and PI3K are frequently hyperactivated or overexpressed in cancers such as cervical cancer and PDAC [35,36]; and (3) although inhibitors of FAK and PI3K have been tested individually in clinical and preclinical settings with variable outcomes [37,38], their combined use has not been extensively evaluated.

Our findings demonstrated that combining FAK and PI3K inhibitors synergistically suppressed the growth of cervical and pancreatic cancer cell lines by enhancing apoptosis and reducing mitosis. When used alone, PI3K inhibitors caused only transient suppression of downstream AKT activity while paradoxically activating FAK signaling in cancer cells. Adding an FAK inhibitor effectively mitigated the PI3K-inhibitor-induced activation of FAK. Additionally, PI3K inhibitors were shown to activate multiple receptor tyrosine kinases (RTKs), highlighting the necessity of FAK inhibition to counteract this compensatory RTK activation. These results underscore the therapeutic potential of dual inhibition of FAK and PI3K in cancer treatment.

## 2. Results

### 2.1. Synergy Between FAK and PI3K Inhibition on the Growth of Cervical and Pancreatic Cancer Cells

**Figure 1:**
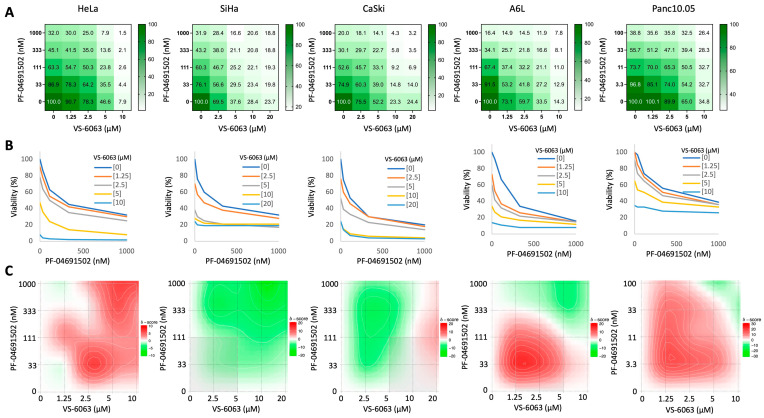
Synergy between PI3K and FAK inhibitors in cervical and pancreatic cancer cell lines. (**A**,**B**) Viability of cancer cell lines measured by CCK-8 assay after treatment with indicated combinations of PF-04691502 and VS-6063 for 48 h. The numbers represent the percentage of viability compared to that of DMSO control calculated from 3 independent experiments. (**B**) Comparison of PF-04691502 viability dose curve at different concentrations of VS-6063. (**C**) Bliss synergy score for PF-04691502 and VS-6063 calculated from (**A**,**B**) using SynergyFinder [39]. Red and green colors correspond to high and low synergy scores, respectively.

We investigated the impact of combined PI3K and FAK inhibition on cancer cell growth. Here we use cervical and pancreatic cancer cell lines due to the frequent activation of PI3K and FAK in these cancers as mentioned above. First, we assessed the IC50 values of PF-04691502 (a PI3K/mTOR dual inhibitor) and VS-6063 (defactinib, a FAK inhibitor) in suppressing the growth of three cervical cancer lines: HeLa, SiHa, and CaSki. In these cell lines, the IC50 was approximately 0.1 μM for PF-04691502 and 5 μM for VS-6063 (Figure S1A). Subsequently, we treated the cells with combinations of varying concentrations of PF-04691502 and VS-6063. While both PF-04691502 and VS-6063 caused dose-dependent suppression of cell growth as single agents, the addition of VS-6063 potentiated the effect of PF-04691502 and vice versa (Figure 1A,B). Similar effects of the two inhibitors were also observed in two pancreatic cancer cell lines, A6L and Panc10.05. An evaluation of the Bliss score revealed strong synergy between PF-04691502 and VS-6063 at certain concentrations in all cell lines except for SiHa (Figure 1C red areas).

**Figure 2:**
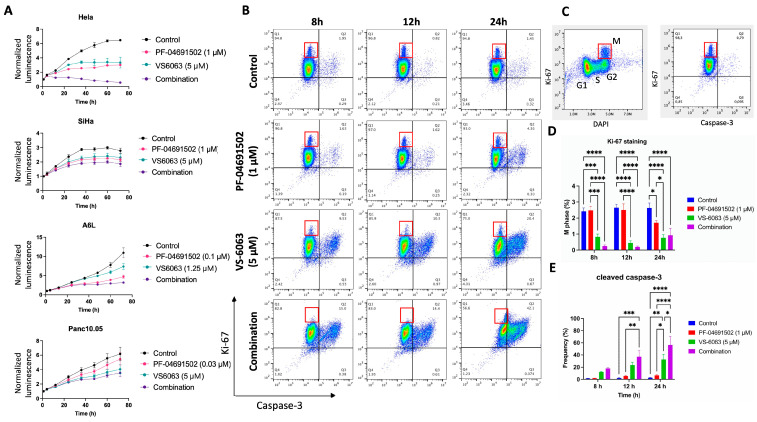
Effects of combined PI3K and FAK inhibition on cell proliferation, apoptosis, and cell cycle. (**A**) Proliferation of cells treated with indicated concentrations of PF-04691502 and VS-6063 tracked using the RealTime-Glo MT Cell Viability Assay (*n* = 6, error bars: s.e.m.). (**B**) Flow cytometry analysis of caspase-3 and Ki-67 staining of HeLa cells treated with indicated concentrations of PF-04691502 and VS-6063 for 8h, 12h, and 24h. (**C**) Example of cell cycle phase determination by Ki-67 and DAPI staining. In (**B**,**C**), red boxes indicate M phase cells. (**D**,**E**) Quantification of M phase (red boxes) and caspase-3 (+) cells in (B) (n = 4, error bars: s.e.m.). Statistical significance was defined using the Tukey method (* *p* < 0.05, ** *p* < 0.01, *** *p* < 0.001 and **** *p* < 0.0001).

To gain insight into the dynamics of cell growth in response to PI3K and FAK inhibitor combinations, we utilized the RealTime-Glo MT Cell Viability Assay to track cell behavior over a 72 h period. For HeLa cells, both PF-04691502 and VS-6063 exhibited cytostatic effects as individual agents but demonstrated cytocidal effects when combined (Figure 2A). In contrast, the effects of inhibitors on SiHa, A6L, and Panc10.05 were mainly cytostatic. To further investigate the impact of the inhibitors on cell growth and death, we treated HeLa cells with inhibitors followed by flow cytometry analysis of Ki-67, caspase-3, and DAPI staining at 8, 12, and 24 h after treatment (Figure 2B and Figure S2). Mitotic (M phase) cells can be identified by a high Ki-67 level and DNA content determined by DAPI staining (Figure 2B,C, red boxes). VS-6063 treatment led to a significant decrease in M phase cells (Figure 2D) and increase in caspase-3 (+) cells (Figure 2E). Interestingly, PF-04691502 by itself had limited effects on cell cycle progression and cell death, but synergized with VS-6063 to reduce M phase cells and increase caspase-3 (+) cells (Figure 2D,E). Together, these findings suggest that PF-04691502 and VS-6063 work together to increase cell death and inhibit cell cycle progression.

### 2.2. Signaling Effects of PI3K and FAK Inhibitors in Cervical and Pancreatic Cancer Cells

**Figure 3:**
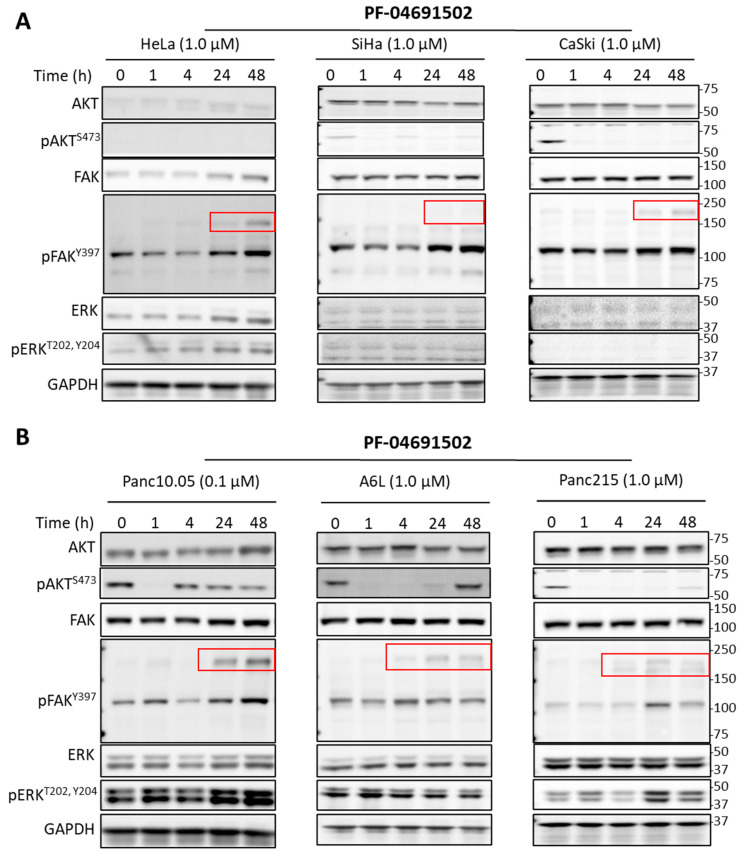
Immunoblotting of cervical (**A**) and pancreatic (**B**) cancer cells treated with PF-04691502. Cells were treated with PF-04691502 for different incubation periods and immunoblotted for total and phosphorylated forms of AKT, FAK, and ERK. GAPDH was used as a loading control. Red boxes indicate the extra bands above the expected FAK size (125 kDa) induced by PF-04691502 treatment.

**Figure 4:**
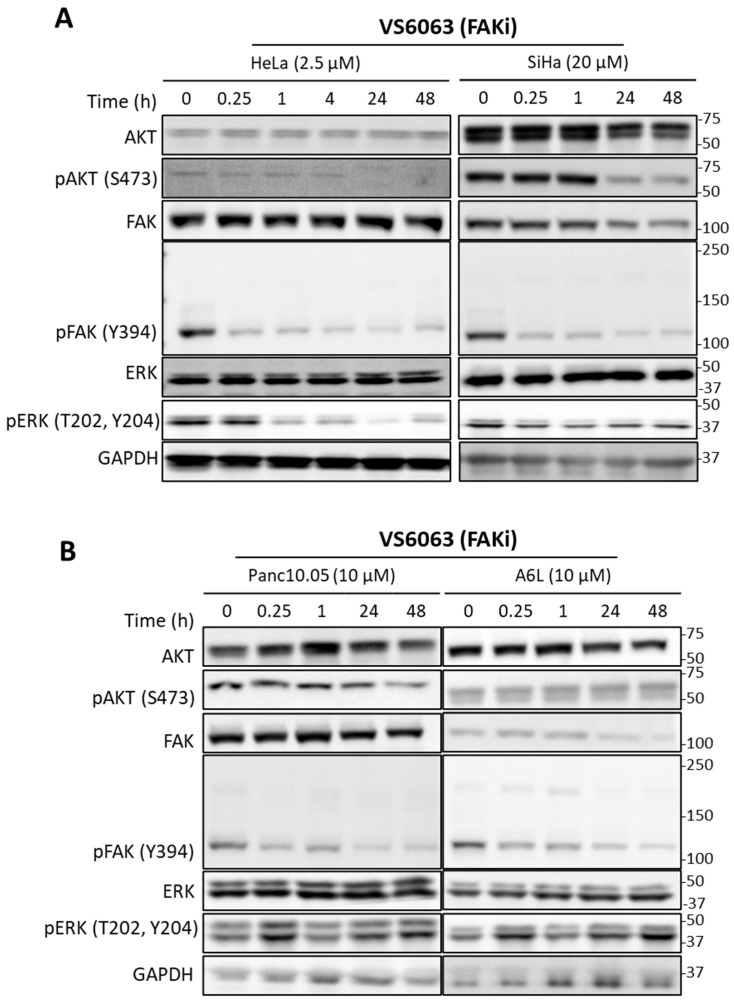
Immunoblotting of cervical and pancreatic cancer cells treated with FAK inhibitors. Cervical (**A**) and pancreatic (**B**) cancer cells were treated with VS-6063, and samples were taken at the indicated time points for immunoblotting of total and phosphorylated forms of AKT, FAK, and ERK, as well as the GAPDH loading control.

To assess the signaling effects of PI3K and FAK inhibitors, we carried out immunoblotting experiments, using phospho-AKT and phospho-FAK as the readout for PI3K and FAK activities. Treatment with PF-04691502 led to the immediate suppression of phospho-AKT in all cancer cell lines (the level was undetectable in HeLa); however, the level rebounded partially at later time points in multiple cell lines (Figure 3A,B). To rule out that the pAKT rebound was due to inhibitor degradation, we collected conditioned media from cells treated with the inhibitor for 0~72 h. When the conditioned media were added to fresh cells, phospho-AKT was inhibited, suggesting that the PI3K inhibitor retained its activity (Figure S3). Thus, reactivation of AKT in the presence of PI3K inhibition was not due to loss of inhibitor potency. In contrast to the transient effect of PF-04691502, VS-6063 caused sustained reduction in phospho-FAK and phospho-AKT in both cervical and pancreatic cell lines (Figure 4).

**Figure 5:**
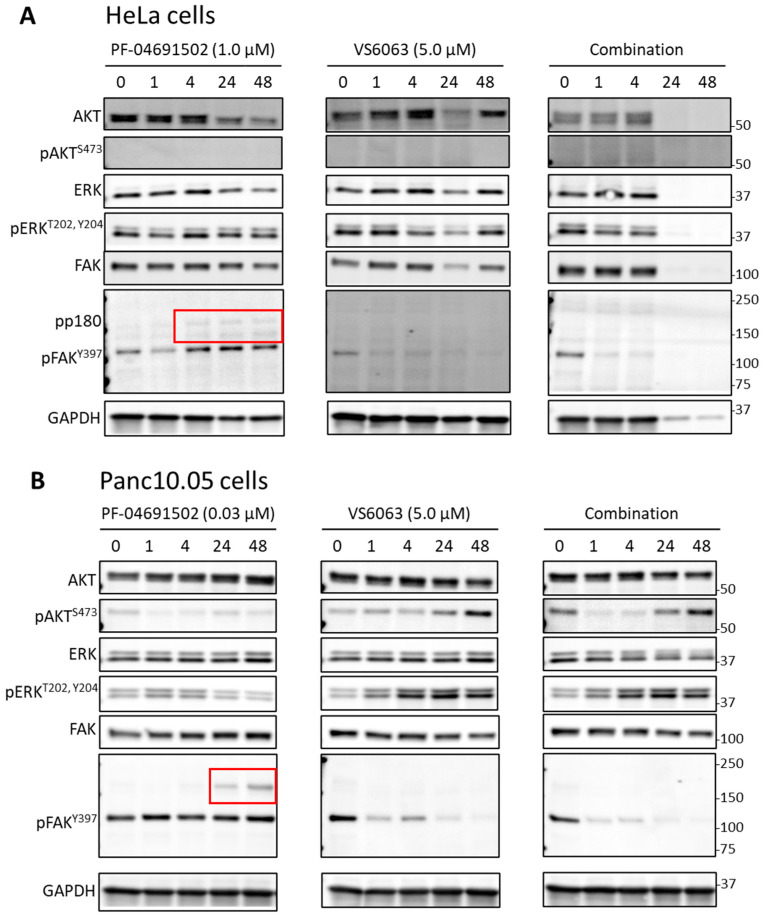
Immunoblotting of HeLa cells treated with PI3K and FAK inhibitors. HeLa cells (**A**) and Panc10.05 (**B**) cells were treated with PF-04691502, VS-6063, or the combination of the two inhibitors. Samples were taken at the indicated time points for immunoblotting of total and phosphorylated forms of AKT, FAK, and ERK, as well as the GAPDH loading control. Red boxes indicate the extra bands above the expected FAK size (125 kDa) induced by PF-04691502 treatment.

Interestingly, PF-04691502 also caused an increase in phospho-FAK in multiple cell lines with varying kinetics over 48 h (Figure 3). The addition of VS-6063 effectively suppressed the FAK activation (Figure 5). This is accompanied by extensive cell death, especially in HeLa cells (Figure 5A, combination 24 and 48 h). We also examined the effect of PF-04691502 and VS-6063 on ERK signaling using phospho-ERK as a readout. However, the response of phospho-ERK showed great variability between experiments (e.g., compare Figure 3A and Figure 5A). This is likely due to the pulsatile nature of ERK activities [25,26], which cannot be captured by sampling at specific time points. Together, these observations suggest that FAK inhibitors may synergize with PI3K inhibitors, suppressing FAK activation induced by the latter.

### 2.3. PI3K Inhibition Induces Activation of Multiple RTKs

**Figure 6:**
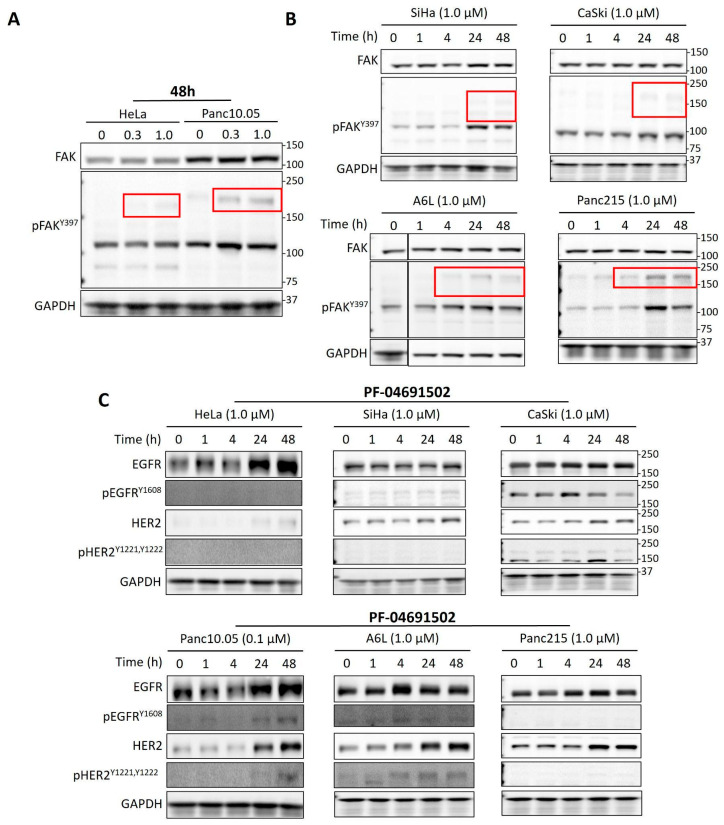
Immunoblotting of cells treated with PI3K inhibitors. (**A**,**B**) Immunoblots of FAK and phospho-FAK for HeLa and Panc10.05 cells treated with DMSO control (0 μM ZSTK474 and time at 0 h) and different concentrations of ZSTK474. (**B**) Immunoblots of FAK and phospho-FAK for cervical (top) and pancreatic (bottom) cancer cells treated with ZSTK474 for the indicated duration. Red boxes indicate the unknown bands found in phospho-FAK immunoblots. Red boxes indicate the extra bands above the expected FAK size (125 kDa) induced by ZSTK474 treatment. (**C**) Immunoblots of EGFR, phospho-EGFR, HER2, and phospho-HER2 for cervical (top) and pancreatic (bottom) cancer cells treated with PF-04691502 for the indicated duration.

Interestingly, cells treated with PF-04691502 showed extra bands above the FAK band (125 kDa) after 24–48 h in the phospho-FAK immunoblot (Figure 3A,B, red boxes). The size of the bands varied among the cell lines, with a molecular weight range of 150~180 kD except for SiHa, in which the size was smaller than 150 kD. Treating cells with ZSTK474, a pan-class I PI3K inhibitor (PI3Ki), induced similar bands in a dose- and time-dependent manner (Figure 6A,B). Since the phospho-FAK antibody is known to cross-react with tyrosine-phosphorylated proteins such as EGFR, we investigated whether the high molecular weight bands induced by PI3K inhibition corresponded to the activation of EGFR and HER2, which have similar molecular weights. Our results showed that treatment with PF-04691502 significantly increased the levels of phospho-EGFR and HER2/phospho-HER2 in Pan10.05, as well as HER2/phospho-HER2 in A6L (Figure 6C). Although EGFR and HER2 levels were also increased in cervical cancer cell lines, they were not accompanied by an increase in the phosphorylated forms of these proteins.

**Figure 7:**
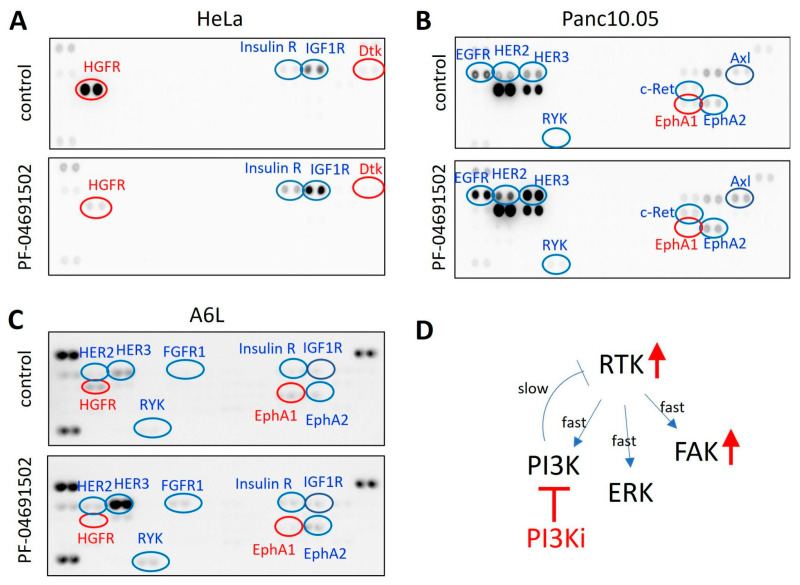
Phospho-RTK array of HeLa, Panc10.05, and A6L cells. (**A**–**C**) Cells were treated with DMSO or PF-04691502 for 48 h, and the phospho-RTK activity was probed using the Proteome Profiler Human Phospho-RTK Array Kit (R&D Systems, ARY001B). Red and green ovals denote spots showing decreased and increased intensity, respectively, after PF-04691502 treatment. (**D**) A working model depicting how PI3K inhibition releases negative feedback from PI3K to RTK, leading to RTK activation and FAK activation. Simultaneous FAK inhibition is required to combat this effect. → indicates activation; ⊣ indicates inhibition.

To comprehensively identify the RTKs potentially activated by PI3K inhibition, we conducted phospho-RTK array analyses on cells before and after 48 h of PI3K inhibition. In HeLa cells, treatment with PF-04691502 resulted in increased phosphorylation of the insulin receptor (IR) and IGF-1R, while phosphorylation of HGFR and Dtk was reduced (Figure 7A). Conversely, in Panc10.05 cells, PF-04691502 treatment led to increased phosphorylation of multiple RTKs, including EGFR, HER2, HER3, AXL, and EphA2 (Figure 7B). Unlike the results observed in HeLa cells, HGFR activity did not decrease in Panc10.05 cells after 48 h of PF-04691502 treatment. An increased phosphorylation of multiple RTKs, most notably HER3, was also noticed in A6L cells after PF-04691502 treatment (Figure 7C). The changes in EGFR and HER2 phosphorylation are consistent with those shown in Figure 6C. We further carried out immunoblotting of HER3/phospho-HER and confirmed the increase in phospho-HER3 in Panc10.05 and A6L cells (Figure S4). Interestingly, there was also a slight increase in phospho-HER3 in HeLa cells, but the overall level was very low, explaining why it was not detected in the phospho-RTK array. Together, these results indicate that PI3K inhibition led to cell-type-dependent changes in multiple RTK activities.

## 3. Discussion

In this study, we explored the idea of targeting the excitability of the Ras signaling network for cancer treatment. We previously identified a positive feedback loop between Ras, PI3K, the cytoskeleton, and FAK that is required for the excitability [23]. Due to the redundancy in signaling networks, we reasoned that an effective blockade of excitability requires simultaneous inhibition of more than one node in the feedback loop. Vertical inhibition of the same pathway has been found to be more effective than blocking a single node in cancer treatment, as exemplified by the combination of BRAF and MEK inhibitors in BRAF mutant melanoma [40,41]. Here, we chose to test the effects of simultaneous FAK and PI3K inhibition on the growth and signaling of cancer cells. Our findings revealed that the combination of FAK and PI3K inhibitors synergistically inhibited the growth of select cervical and pancreatic cancer cell lines by promoting apoptosis and suppressing mitosis.

FAK is commonly overexpressed in various cancer types [35,36,42]. Its inhibition has been shown to reduce cervical cancer xenograft growth and metastasis [43], suggesting FAK as a promising cancer target. However, as a single agent, FAK inhibitors have demonstrated limited efficacy. FAK has also been implicated in resistance to targeted therapies, highlighting the potential for enhanced effectiveness when combined with other inhibitors [37,42].

PI3K signaling, critical for cell growth, proliferation, and survival, is an appealing cancer treatment target. Despite this, PI3K inhibitors have shown limited success in clinical trials due to the development of resistance, narrow therapeutic windows, and frequent treatment-related side effects [44,45]. Resistance to PI3K inhibitors may arise from RTK–ligand interactions driven by autocrine production, paracrine contributions from tumor stroma, or systemic production [46,47]. To address these challenges, combining PI3K inhibitors with RTK inhibitors or blocking antibodies has been proposed as a potential strategy [48,49,50].

Extensive crosstalk exists between FAK and PI3K signaling pathways [51]; however, studies on their simultaneous targeting are limited. Our findings demonstrate the synergy between PI3K and FAK inhibitors in cervical and pancreatic cancer cell lines. A prior study also reported potential synergy between PI3K and FAK inhibitors in lung cancer cells with reduced PTEN [52]. Mechanistically, we observed that PI3K inhibition led to FAK activation. This effect is likely due to PI3K inhibition inducing the activation of multiple RTKs, including IR and IGF-1R in cervical cancer cells and EGFR, HER2, HER3, AXL, and EphA2 in pancreatic cancer cells. Since FAK is a well-documented downstream effector of RTKs [53,54], the activation of FAK by PI3K inhibition may result from RTK activation (Figure 7D), and simultaneous inhibition of PI3K and FAK is thus required to effectively suppress cancer cell growth.

We observed that total EGFR levels were elevated in HeLa and Panc10.05 cells treated with a PI3K inhibitor, while increased total HER2 levels were observed in HeLa, Panc10.05, A6L, and Panc215 cells (Figure 6C). These findings align with previous reports that PI3K inhibition can relieve negative feedback mechanisms that suppress RTK expression and activity. One well-characterized mechanism involves the FOXO family of transcription factors. Under conditions of active PI3K-AKT signaling, FOXO proteins are phosphorylated by AKT, leading to their sequestration in the cytoplasm and the inhibition of their transcriptional activity. When PI3K is inhibited and AKT activity decreases, FOXO proteins translocate to the nucleus and activate the transcription of several RTKs, including HER3, IGF-1R, and INSR, thereby reinstating upstream signaling and limiting the efficacy of PI3K inhibitors [55,56,57]. This feedback loop represents an adaptive survival mechanism through which cancer cells attempt to bypass PI3K blockade.

In addition to transcriptional regulation, our data suggest that RTK activation following PI3K inhibition may also involve post-translational mechanisms. For example, in Panc10.05 cells, PI3K inhibition had little effect on the total HER3 level but significantly increased phosphor-HER3 levels (Figure S4). One possibility is that the inhibition of PI3K could alter the balance between RTK kinase and phosphatase activities, leading to enhanced RTK phosphorylation. However, the molecular mechanism of this process requires further investigation.

Together, our findings suggest that dual inhibition of FAK and PI3K is a promising therapeutic strategy for cervical and pancreatic cancers, and possibly other tumors driven by hyperactivation of the Ras/PI3K signaling network. However, the variability in sensitivity to FAK and PI3K co-inhibition among different cancer cell lines highlights the need for predictive biomarkers to guide patient selection. While some cell lines exhibited strong synergy between inhibitors, others showed only modest or no benefit, suggesting that intrinsic genetic, epigenetic, or signaling differences influence treatment response. Key factors such as baseline PI3K/FAK activation levels, RTK expression profiles, and compensatory feedback loops may play a crucial role in determining sensitivity.

One limitation of this study is that the findings are based on in vitro models. To establish clinical relevance, in vivo studies are necessary to assess the efficacy of PI3K and FAK inhibition in animal models. Key aspects of validation include the use of xenograft and patient-derived xenograft (PDX) models to evaluate tumor growth inhibition, apoptosis induction, and metastasis suppression. Additionally, combining inhibitors may increase the risk of side effects or toxicity, necessitating pharmacokinetic and toxicity studies to assess drug distribution, half-life, and tolerability. Given the dose-limiting toxicities commonly associated with PI3K inhibitors, the potential adverse effects of their combination with FAK inhibition—particularly on the immune system and vascular integrity—should be closely monitored.

A critical challenge in targeted cancer therapy is patient stratification. Since PI3K and FAK inhibitors have demonstrated limited efficacy as monotherapies in clinical trials, identifying biomarkers that predict sensitivity to their combination is essential. Given the observed compensatory RTK activation, future studies should also explore whether co-targeting FAK/PI3K alongside RTK inhibitors (e.g., HER3 or IGF-1R inhibitors) could further enhance therapeutic efficacy and overcome adaptive resistance mechanisms.

## 4. Materials and Methods

### 4.1. Cell Lines

HeLa, SiHa, and CaSki cell lines were purchased from ATCC. Panc10.05, A6L and Panc215 cells were kindly provided by the lab of Laura Wood (JHU). The cells were cultured at 37 °C with 5% CO_2_ in DMEM high-glucose medium (Gibco, Waltham, MA, USA, #11965092) supplemented with 10% FBS (Corning, Corning, NY, USA, 35-010-CV), 1 mM sodium pyruvate (Gibco, Waltham, MA, USA, #11360070), and 1X nonessential amino acids (Gibco, Waltham, MA, USA, #11140050). CaSki cells were grown at 37 °C with 5% CO_2_ in RPMI-1640 high-glucose medium (Gibco, Waltham, MA, USA, #11965092) supplemented with 10% FBS, 1 mM sodium pyruvate, and 1× nonessential amino acids.

### 4.2. Chemical Reagents

Stocks of 10 mM PF-04691502 (Sigma-Aldrich, St. Louis, MO, USA, PZ0235), 5 mM ZSTK474 (Selleckchem, Houston, TX, USA, S1072), and 50 mM VS-6063 (defactinib; Selleckchem, Houston, TX, USA, S7654) were prepared by dissolving the chemicals in DMSO. All drug stocks were stored at −20 °C.

### 4.3. Cell Viability Assay

The viability of cells after drug treatment was performed by Cell Counting Kit-8 (CCK-8, Dojindo, Rockville, MD, USA, CK04-20) according to the manufacturer’s instructions. Briefly, 3000 cells were seeded in a 96-well plate and incubated at 37 °C with 5% CO_2_ overnight before being exposed to drugs. After a designated period of incubation, the CCK-8 reagent was directly added into each well and incubated with cells for another 3h. The absorbance of each well was analyzed by an ELISA reader (800 TS absorbance reader, Agilent BioTek, Santa Clara, CA, USA) equipped with a 450 nm filter.

### 4.4. Analysis of Synergy

Bliss synergy scores were calculated using SynergyFinder (version 2.0) [39]. Briefly, cell viability data were formatted according to the example table provided on the SynergyFinder website (https://synergyfinder.fimm.fi, accessed on 26 August 2021) and then uploaded to the platform. The four-parameter logistic regression (LL4) model was selected for curve fitting, with outlier detection enabled. The Bliss model was used to assess the effects of the drug combinations.

### 4.5. RealTime-Glo MT Cell Viability Assay

Cell viability was monitored in real time using the RealTime-Glo MT Cell Viability Assay kit (Promega, Madison, WI, USA, G9713) following the manufacturer’s instructions. Briefly, 3000 cells were plated in a 96-well white opaque plate and incubated overnight. The next day, the medium was replaced with phenol red-free medium containing either the vehicle (DMSO), PF-04691502, VS-6063, or a combination of both drugs. The 1000× MT Cell Viability Substrate and 1000× NanoLuc^®^ Enzyme were diluted to 2× in the medium and added to each well in equal volumes. Luminescence intensity, indicating cell viability, was measured using a BMG LABTECH FLUOstar Omega Microplate Reader (Cary, NC, USA).

### 4.6. Cell Cycle, Cell Growth and Apoptosis Analysis by Flow Cytometry

Flow cytometry was used to examine DAPI for cell cycle analysis, Ki-67 for cell proliferation, and cleaved caspase 3 expression for apoptosis following drug treatment. Cells were fixed with 4% formaldehyde and permeabilized using ice-cold methanol. The cells were then incubated with DAPI (10 mg/mL, 1:1000), mouse anti-human Ki-67 (Cell Signaling Technology, Danvers, MA, USA, #9449, 1:400), and rabbit anti-human cleaved caspase 3 antibodies (Cell Signaling #9661, 1:800) at room temperature for 1 h. Anti-mouse IgG DyLight 594 (Invitrogen #35511, 1:100) and anti-rabbit IgG DyLight 488 (Invitrogen, Waltham, MA, USA #35553, 1:100) were used to detect anti-Ki-67 and anti-cleaved caspase 3 antibodies, respectively. The experiments were carried out on a CytoFLEX Flow Cytometry (Beckman Coulter, Brea, CA, USA), and data were analyzed with FlowJo v10 from BD Biosciences (Franklin Lakes, NJ, USA).

### 4.7. Immunoblotting

The following primary antibodies were purchased from Cell Signaling Technology: FAK (#13009, 1:1000), phospho-FAK (#8556, 1:500), AKT (#2920, 1:1000), phospho-AKT (#4060, 1:1000), ERK (#9107, 1:1000), phospho-ERK (#9101, 1:1000), EGFR (#4267, 1:1000), phospho-EGFR (#3777, 1:1000), HER2 (#2165, 1:1000), phospho-HER2 (#2243, 1:1000), HER3 (#12708, 1:1000), phospho-HER3 (#4791, 1:1000), and GAPDH (#2118, 1:2000). Secondary antibodies used were Donkey anti-Rabbit Alexa Fluor^TM^ 647 (ThermoFisher, Danvers, MA, USA, A-31573, 1:5000) and Donkey anti-Mouse Alexa FluorTM 647 (ThermoFisher, Danvers, MA, USA, A-31571, 1:5000).

Immunoblotting was performed as described in our previous publication [23]. Briefly, cells were harvested and lysed in 1X RIPA buffer (Cell Signaling, #9806) containing 1× protease inhibitor cocktail (Roche, Indianapolis, IN, USA, #11873580001) and 1X phosphatase inhibitor (Sigma-Aldrich, St. Louis, MO, USA, #P5726). Cell lysates were collected after centrifugation. After mixing with sample buffer and boiling at 95 °C for 5 min, proteins were separated by SDS-PAGE using 4–20% Criterion™ TGX™ Precast Midi Protein Gels (Bio-Rad, Hercules, CA, USA, #5671094) and then transferred to a low-fluorescence-background PVDF membrane (Sigma-Aldrich, St. Louis, MO, USA, #IPFL00005) in an ice bath at 80V for 1h. Membranes were incubated with primary antibodies overnight followed by incubation with secondary antibodies in 5% BSA in TBST at room temperature for 1h. Images were taken by a Pharos Molecular Imager (Bio-Rad, Hercules, CA, USA) and analyzed using ImageJ 1.54f/Fiji 2.14.0 [58,59]. The background was subtracted from the intensities of individual protein bands. Phosphoprotein levels were normalized to either GAPDH or the total level of the corresponding protein.

### 4.8. Phospho-RTK Array

Receptor tyrosine kinase (RTK) activities in cells following drug treatment were analyzed using the Proteome Profiler Human Phospho-RTK Array Kit (R&D Systems, Minneapolis, MN, USA, ARY001B) according to the manufacturer’s instructions. After treatment for the specified durations, cells were harvested and lysed using the lysis buffer provided in the kit (R&D Systems, #895943). The cell lysates were centrifuged at 14,000× *g* for 5 min at 4 °C, and the supernatant was collected. The array membrane was first soaked in the blocking buffer at room temperature for 1 h and then incubated with the lysate samples overnight at 4 °C. Following washes with the wash buffer, the membrane was incubated with secondary antibodies at room temperature for 2 h. The resulting images were captured using the ChemiDoc™ Touch Imaging System (Bio-Rad).

### 4.9. Statistical Analysis

Experiments were repeated on different days with the number of repeats (n) indicated in the figure legends. Statistical analyses were performed via two-way ANOVA using GraphPad Prism (version 10.3.1) software. Mean ± s.e.m. (standard error of the mean) was reported, and statistical significance was defined using the Tukey method (* *p* < 0.05, ** *p* < 0.01, and *** *p* < 0.001).

## 5. Conclusions

In conclusion, our findings demonstrate a synergistic effect between PI3K and FAK inhibitors in suppressing cancer cell growth and offer insights into the potential mechanisms underlying this effect. This highlights the potential of combining these inhibitors as a therapeutic strategy against certain malignancies. Our study is limited to the in vitro and molecular effects of PI3K and FAK inhibitors, and further investigation is required to assess whether these findings translate to in vivo models and clinical settings. Additionally, cancer cells exhibit varying sensitivities to these combinations, emphasizing the need to identify and establish biomarkers of sensitivity. Such biomarkers would be invaluable in guiding patient selection, enabling personalized medicine, and maximizing the efficacy of this combination therapy.

## Data Availability

The raw data supporting the conclusions of this article will be made available by the authors on request.

## Supplementary Materials

The following supporting information can be downloaded at: https://www.mdpi.com/article/10.3390/ijms26073040/s1.
