# Supplementary material for: Targeting Signaling Excitability in Cervical and Pancreatic Cancer Cells Through Combined Inhibition of FAK and PI3K
Source: Int J Mol Sci. 2025 Mar 26;26(7):3040. doi: 10.3390/ijms26073040 (PMC11988430; doi:10.3390/ijms26073040)
Supplement: Supplementary file 1 [file ijms-26-03040-s001.zip › ijms-3538982-supplementary.pdf]

Supplementary Materials

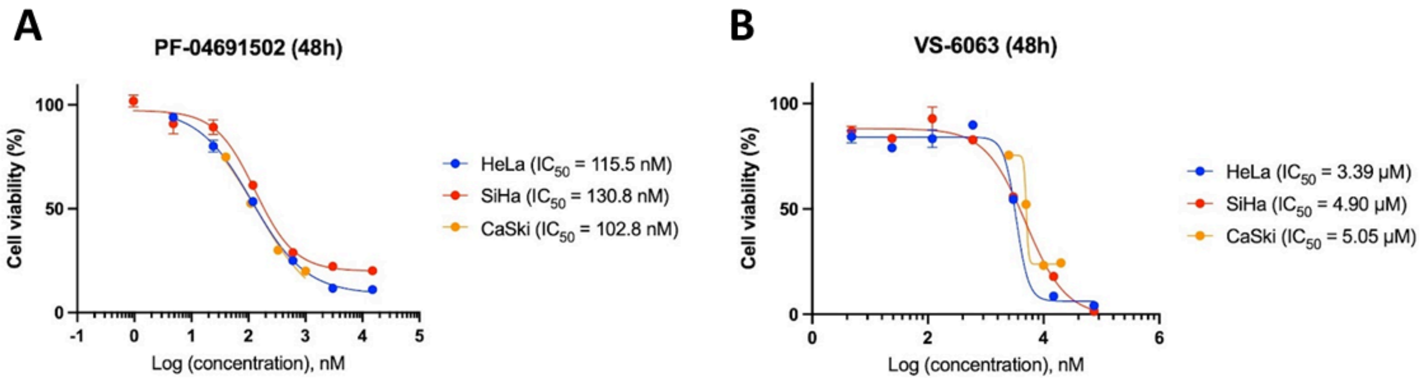

Figure S1. IC50 of PF-04691502 (A) and VS-6063 (B) in cervical cancer cell lines.

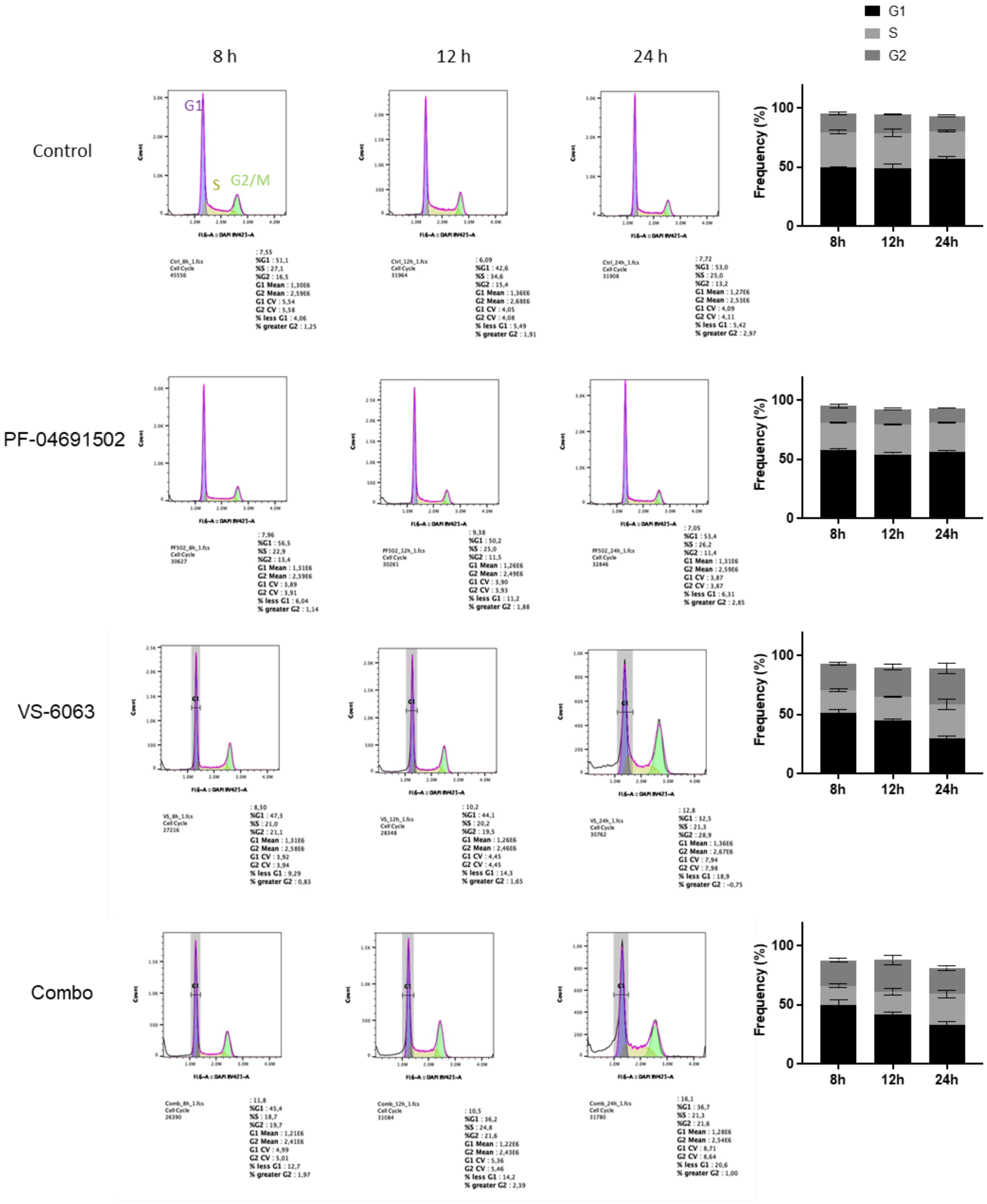

Figure S2. Cell cycle analysis of HeLa cells by flow cytometry of DAPI staining.

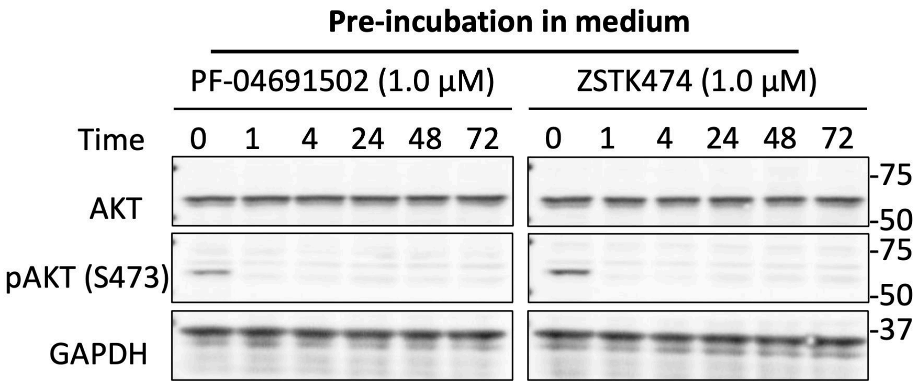

Figure S3. Stability of PI3K inhibitors. A6L cells were treated with the indicated inhibitors PF-04691502 or ZSTK474 for different periods. The medium was collected and transferred to another new plate of A6L cells. After 1 h incubation, cells were harvested for immunoblotting with the indicated antibodies.

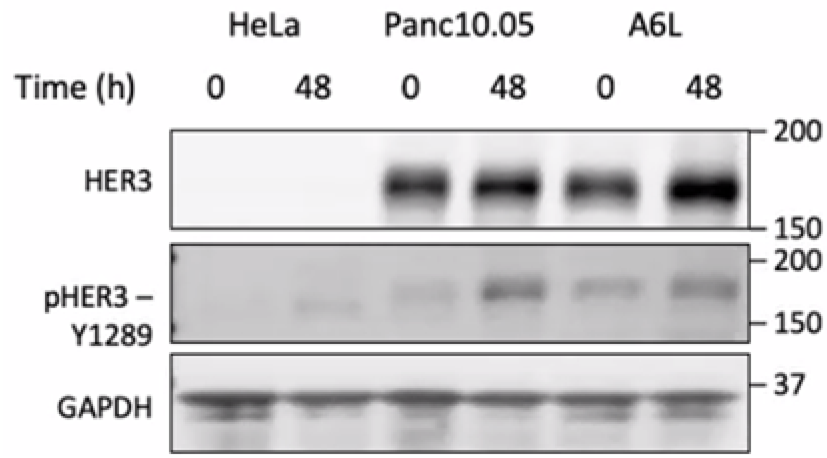

Figure S4. Immunoblotting of HeLa, Panc10.05, and A6L cells treated with PF-04691502.
Immunoblots of HER3 and phospho-HER3 for HeLa, Panc10.05, and A6L cells before and after treated with PF-04691502 for 48 hours. .
